# Conservative management versus open reduction and internal fixation for mid-shaft clavicle fractures in adults - The Clavicle Trial: study protocol for a multicentre randomized controlled trial

**DOI:** 10.1186/1745-6215-12-57

**Published:** 2011-02-28

**Authors:** Umile Giuseppe Longo, Sughran Banerjee, Julie Barber, Andrew Chambler, Carlos Cobiella, Steven Corbett, Mark Crowther, Steven Drew, Andrea Francis, Marcus Lee, Nicholas Garlick, Iain Packham, Yemi Pearse, Andrew Richards, Chris Roberts, Duncan Tennent, Emily Tims, Philip Michael Ahrens

**Affiliations:** 1 Department of Orthopaedic and Trauma Surgery, Campus Bio-Medico University, Via Alvaro del Portillo, 200, 00128 Rome, Italy; 2 Department of Orthopaedics, Royal Free Hampstead NHS Trust, Pond Street, London, NW3 2QG, UK; 3 University College London Hospital NHS Foundation Trust, 250 Euston Road, London NW1 2PG, UK; 4 Yeovil District Hospital NHS Foundation Trust, Higher Kingston, Yeovil, Somerset BA21 4AT, UK; 5 Department of Orthopaedics, Guy's and St Thomas' NHS Foundation Trust, Westminster Bridge Road, London SE1 7EH, UK; 6 St George's Healthcare NHS Trust, Blackshaw Road, Tooting, London, SW17 0QT, UK; 7 North Bristol NHS Trust, Frenchay Hospital, North Bristol NHS Trust, Frenchay, Bristol, BS16 1LE, UK; 8 The Ipswich Hospital NHS Trust, Heath Road, Ipswich, Suffolk, IP4 5PD, UK; 9 University Hospital, Clifford Bridge Road, Coventry CV2 2DX, UK

## Abstract

**Background:**

Clavicle fractures account for around 4% of all fractures and up to 44% of fractures of the shoulder girdle. Fractures of the middle third (or mid-shaft) account for approximately 80% of all clavicle fractures. Management of this group of fractures is often challenging and the outcome can be unsatisfactory. In particular it is not clear whether surgery produces better outcomes than non-surgical management. Currently there is much variation in the use of surgery and a lack of good quality evidence to inform our decision.

**Methods/Design:**

We aim to undertake a multicentre randomised controlled trial evaluating the effectiveness and safety of conservative management versus open reduction and internal fixation for displaced mid-shaft clavicle fractures in adults. Surgical treatment will be performed using the Acumed clavicle fixation system. Conservative management will consist of immobilisation in a sling at the side in internal rotation for 6 weeks or until clinical or radiological union. We aim to recruit 300 patients. These patients will be followed-up for at least 9 months. The primary endpoint will be the rate of non-union at 3 months following treatment. Secondary endpoints will be limb function measured using the Constant-Murley Score and the Disabilities of the Arm, Shoulder and Hand (DASH) Score at 3 and 9 months post-operatively.

**Discussion:**

This article presents the protocol for a multicentre randomised controlled trial. It gives extensive details of, and the basis for, the chosen methods, and describes the key measures taken to avoid bias and to ensure validity.

**Trial Registration:**

United Kingdom Clinical Research Network ID: 8665. The date of registration of the trial is 07/09/2006. The date the first patient was recruited is 18/12/2007.

## Background

### Rationale for the trial

Clavicle fractures account for around 4% of all fractures [1] and up to 44% of fractures of the shoulder girdle [2,3]. Fractures of the middle third (or mid-shaft) account for approximately 80% of all clavicle fractures [2,3].

Management of mid-shaft clavicular fractures is often challenging and the outcome can be unsatisfactory. In particular it is not clear whether surgery produces better outcomes than non-surgical management. Traditionally mid-shaft clavicular fractures have been managed conservatively, even when substantially displaced [4].

Recent literature has highlighted the high non-union rate in displaced mid-shaft clavicular fractures, with a non-union rate up to 15% [5-7]. Furthermore, there is some evidence that conservative management affects the outcome in terms of upper limb function [8-10] and that treatment of non-unions produces inferior results [11,12]. Few comparative studies of surgical versus non-surgical treatment for mid-shaft clavicle fractures are available, and contradictory results have been obtained [13-15].

In a randomised controlled trial, though patients with mid-shaft clavicle fractures had higher functional scores at short-term follow-up after internal fixation, functional scores were similar at 6 months and 1 year. In addition, internal fixation with a modified Hagie pin was associated with a higher complication rate [13].

In a multicentre, prospective clinical trial [16], 132 patients with a displaced mid-shaft fracture of the clavicle were randomized to either operative treatment with plate fixation (67 patients) or nonoperative treatment with a sling (65 patients). Operative fixation of a displaced fracture of the clavicular shaft resulted in improved functional outcome and a lower rate of mal-union and non-union compared with nonoperative treatment at one year of follow-up. Hardware removal remained the most common reason for repeat intervention in the operative group.

A formal cost-effectiveness analysis based on the same prospective, randomized, controlled trial [14] proposed that the cost-effectiveness of open reduction and internal fixation (ORIF) after acute clavicle fracture depended on the durability of functional advantage for ORIF compared with nonoperative treatment. When functional benefits persisted for more than 9 years, ORIF had a favourable value compared with many accepted health interventions.

Two 2009 Cochrane reviews [1,11] on the management of middle third clavicle fractures concluded that there is insufficient evidence from randomised controlled trials to determine which methods of conservative [11] and surgical [1] treatment are the most appropriate for middle third clavicle fractures.

A 2010 systematic review [17] concluded that there is moderate evidence that operative treatment for mid-shaft clavicle fractures results in a lower rate of fracture non-union and improved patient-oriented outcome compared to non-operative treatment. However, because union rates are generally high and there are complications which are unique to surgical intervention, risks have to be considered before a decision on treatment is made. The most important risk factors for non-union are major displacement and fracture comminution. Of all surgical procedures the best evidence of efficacy is presently available for plate fixation and elastic stable intramedullary nailing [17].

### Trial aim

The aim of this multicentre randomised controlled trial (RCT) is to compare the safety and effectiveness of conservative and operative management of mid-shaft fractures of the clavicle in adults. The primary outcome will be the non-union at 3 months from injury. Secondary outcomes will be clinical scoring systems assessing pain, mobility, strength and function at 3 and 9 months follow-up.

Our null hypothesis is no difference in the non-union rate at 3 months from injury and in scoring systems at 3 and 9 months follow-up between the 2 groups.

## Methods/Design

### Study Design

This is a multicentre randomised controlled trial comparing safety and effectiveness of conservative management versus ORIF of mid-shaft clavicle fractures.

### Setting

Patients will be recruited from the following centres:

Royal Free Hospital NHS Trust, University College Hospitals NHS Foundation Trust, St Thomas' and Guy's Hospitals NHS Foundation Trust, St George's Hospital NHS Trust, Yeovil District Hospital NHS Foundation Trust, North Bristol NHS Trust, University Hospitals Coventry and Warwick NHS Foundation Trust, The Ipswich Hospital NHS Trust.

### Endpoints

The primary endpoint will be the rate of non-union at 3 months following treatment. Non-union is defined as lack of radiographic healing at 3 month follow up [16,18].

Secondary endpoints will be limb function measured using the Constant-Murley Score [19] and the Disabilities of the Arm, Shoulder and Hand (DASH) Score [20] measured at 3 and 9 months post-operatively.

### Ethical Considerations

#### Ethical approval

Ethical Approval has been obtained from the UK National Research Ethics Service, Charing Cross Hospital Ethics Committee (for multicentre trials) Reference number 06/Q0411/prior to commencement of this study. Local Ethics Committee approval for each unit involved in the trial will also be obtained.

#### Consent Procedures

Informed consent will be obtained from the patient prior to randomization.

Eligible patients will be provided with a patient information sheet and have the opportunity for discussion with a principal investigator at their first orthopaedic attendance. Patients will have up to a 24-hour period in which to seek further advice and reflect before inclusion.

#### Inclusion criteria

• Age 18-65 years

• Mid-shaft fracture of clavicle

• Robinson Classification 2B1 and 2B2 [21]

• Displaced mid-shaft fracture, butterfly fragment +/- comminution on radiographs

• Medically fit to undergo surgery (ASA grade 1-3)

#### Exclusion criteria

• Patient refusal

• Medically unfit (ASA Grade 4/5)

• All other clavicle fractures

• Established non-union from previous fracture

• Previous fractures around the clavicle

• Previous operations to shoulder or clavicle

• Metabolic bone disease

• Clinically important neuro-muscular upper limb disability.

#### Recruitment

Patients will be identified at attendance to the accident and emergency department at each hospital. They will be referred to fracture clinic, as this is the usual practice, and identified as eligible for the trial by the consultant surgeon in charge of the clinic.

It is estimated that in each centre approximately 2 patients per month will meet the inclusion criteria. It is also estimated that 80% of these patients will consent to be involved in the trial. Loss to follow-up will be small until 3 months, though for final follow-up and assessment is estimated a further 10% may be lost to follow-up.

#### Randomisation

Randomisation lists will be computer generated. Lists will be stratified by centre and blocked to ensure similar numbers of patients are allocated to the treatment groups within each centre. Allocations will be concealed in sequentially numbered opaque envelopes held at each site. 1 envelope will be opened for each patient. Eligible patients will be provided with a patient information sheet and consented using a trial consent form prior to randomisation.

#### Blinding

Because of the nature of the study it will not be possible to blind the patients or clinicians involved.

#### Baseline data collection

Baseline data will be collected on all patients before consent. These will include patient demographics, side of injury, mechanism of injury, fracture classification [21], ASA grade and whether the patient is a smoker. These data will be used to assess the balance in characteristics achieved by randomisation and also to compare the eligible group who declined to participate with the randomised group.

#### Operative Details

All procedures will be performed in an orthopaedic theatre under antibiotic cover according to local microbiology protocols in each centre. General anaesthetic will be used for all patients with or without supplementary interscalene blockade. All surgical procedures will be performed by one of the orthopaedic consultants named in the protocol or by their specialist registrar/research fellow under consultant supervision. All the patients enrolled in the study will be treated in a standardised way.

An infraclavicular incision will be used and a myo-periosteal flap elevated from the fracture segments. Fixation will be performed using the Acumed clavicle fixation system (Hillsboro, Oregon), consisting of a pre-contoured titanium plate with 2.7 mm or 3.5 mm screws. Image intensification will be available for all cases and hard copies will be obtained at the end of each procedure. Following wound closure the affected arm will be placed in an arm sling. Mobilisation and rehabilitation will be identical to the non-operative group (see below).

Data from the procedure will be documented on the operation note, including any peri-operative complications or deviations from the standard technique.

#### Non-operative Treatment

The arm on the fractured side will be immobilised in a sling at the side in internal rotation for 6 weeks or until clinical or radiological union. Pendulum and elbow exercises will be allowed the first day presenting in fracture clinic. Patients will be allowed to remove the sling for short periods to wash, dress, write, eat and use a keyboard as soon as comfort allows. Short-lever active and active-assisted exercises below shoulder height are allowed as tolerated. Active mobilisation above the horizontal and cross-arm adduction will be commenced after 6 weeks.

#### Post operative care

The same rehabilitation protocol will be used in patients undergoing surgical management, starting on the first day post-operatively.

#### Time-points

All patients will be followed up at 2 weeks, 6 weeks, 3 months and between 9 and 12 months from randomisation.

Patients will be given follow up dates at each appointment and a supplementary reminder letter will be sent to all patients before their 9 month appointment.

#### Assessment

For all subjects, radiographs will be performed at the 2 week, 6 week and 3 month follow-up. The 2 week appointment will involve a clinical assessment to exclude early complications related to the fracture or surgery, and a routine radiograph to evaluate the fracture position. The 6 week appointment will involve a clinical assessment of fracture healing and a routine radiograph to assess union. Radiological union will be assessed by the principal investigator at each site. If the fracture is radiographically united at 6 weeks no radiographs will be performed at the 3 month appointment. In cases where there is doubt as to the degree of union at 3 months the radiographs will be reviewed by a principal investigator from another site. Clinical data of union including fracture mobility, tenderness and pain will also be obtained at each follow-up. The x-rays of the first 40 subjects' will be reviewed by an independent, blinded radiologist, once the principal investigator has judged the fracture to have united or be un-united. If there will be a discrepancy of opinion greater than 2% (1 patient), this process will be continued for all trial patients. For those radiographs where there is a discrepancy of opinion, the Chief Investigator will review the case and a majority consensus opinion will be gained from 3 Principal Investigators.

The Constant-Murley [19] and Disability Arm Shoulder and Hand score (DASH) [20] scoring systems will be performed at the 3 month and 9 month reviews. The Constant-Murley score [19] is a practitioner completed objective score on a scale of 100 points divided into section for pain, activity, range of movement and strength and is measured for both arms. The Constant-Murley Score is one of the most used shoulder evaluation tool. This scoring system includes both physical examination tests and subjective evaluations by the patients allowing a functional assessment of the shoulder independently by the specific type of disorder. The Constant-Murley Score is divided into 4 subscales: Pain (15 points); Activities of daily living (ADL) (20 points); Range of Motion (ROM) (40 points) and Strength (25 points). The Pain and the ADL scales are self-reported by patients: in the first version of the Constant-Murley Score, the pain scale was scored as none (15 points); mild (10 points); moderate (5 points) and severe (0 points). In the revised version of 2008, the pain scale is scored by a VAS. The ADL score is divided into 4 items: Sleep, 2 points; Work and Recreational Activities/Sport 4 points each; Positioning the Hand in the Space, 10 points. ROM is evaluated as the active elevation of the arms on the sagittal and lateral planes and the internal and external rotation of the shoulders, 10 points each. Finally, strength is evaluated as the number of pounds of pull that the patient can resist in abduction to a maximum of 25 points. The total possible score is 100 points, indicating an asymptomatic and healthy person, while the worst score is 0 points. The validity and responsiveness of Constant-Murley Score were evaluated in successive studies [22]. Evaluating construct validity, the Constant-Murley Score showed strong correlation with other scales. Evaluating reliability, ICCs varied from 0.84 to 0.87.

The DASH score [20] is a patient completed subjective score with 30 analogue scale responses producing a score between 0 and 100 points. The DASH score is a standardized questionnaire that assesses the symptoms and functional status in people with different upper extremity musculoskeletal disorders. The questionnaire consists of three sections: Symptoms, Sport and Music, Work. The first section is composed by 30 items and evaluates symptoms and functional status at the level of disability. The second and third sections are an optional module of four items for Sport and Music and four items for Work. Each item is scored with a five point scale: 1 = no difficulty; 2 = mild difficulty; 3 = moderate difficulty; 4 = severe difficulty; 5 = unable. The result of each module is summed and transformed to obtain the DASH score ranging, for each section, from 0 (no disability) to 100 (severe disability). Relatively to Internal Consistency the DASH has shown in multiple tests to have a high Cronbach's alpha (0.97); the responsiveness of the questionnaire (to self-rated or expected change) was comparable with or better than that of the joint-specific measures in the whole group and in each region [23].

An independent physiotherapy practitioner not involved in patient's surgical care or rehabilitation program will administer both assessment tools. There will a different examinator in each centre, who will be trained for the purpose of this study. The Constant score will be performed in a standardized way, using the same dynamometer in all the centres.

In the event of a patient developing a non-union at 12 weeks or more, he/she will exit the trial and be offered surgical management. Data regarding treatment offered and pursued will be collected.

#### Adverse Events or Complications

An adverse event or complication will be defined as any event that necessitated another operative procedure or additional medical treatment. Non-union will be defined as the lack of radiographic healing with clinical evidence of pain and motion at the fracture site at 3 months. Symptomatic mal-union will be defined as union of the fracture in a shortened, angulated, or displaced position with weakness, easy fatigability, pain on overhead activity, neurologic symptoms, and shoulder asymmetry with a completed or planned corrective osteotomy.

Complex regional pain syndrome will be diagnosed by the presence of dysaesthetic pain and hyperaesthesia extending into the hand of the involved limb, vasomotor changes, skin atrophy, and diffuse osteopenia [24].

### Sample size

Based on a comparison of the percentages of patients with a non union at 12 weeks following treatment, it is estimated that 141 patients will be required in each treatment group to detect at least a reduction in percentages from 15% [6] to 5% non union with 80% power and a significance level of 5% [25]. A 4% non union rate has been quoted in the literature [26], however for the purposes of this calculation we have used a 5% as a maximum acceptable clinical failure rate. By randomising 300 patients (150 per group) the study will be adequately powered with allowance for loss to followup. Recruitment of 300 subjects will also ensure at least 80% power to detect clinically important effects for secondary outcomes. For the DASH score this will allow detection of a 3.3 point difference in mean scores with a significance level of 5% (assuming a standard deviation of 10) [20]. For the Constant-Murley score a 5 point difference in mean scores will be detectable at a 5% significance level (assuming a standard deviation of 14.5) [19]. Calculations have assumed approximate Normality of the scores [25].

It is estimated that 13 patients per month will be recruited to the study. This would lead to a total period of recruitment for the trial of 23 months.

### Data Analysis

The success of randomisation will be made in a visual comparison of the baseline characteristics of those randomised to the two groups: centre, age, gender, side of injury, mechanism of injury, fracture classification [21] and ASA grade.

The representativeness of the groups randomised to the trial will be examined by comparing their baseline characteristics with those who were eligible but refused to participate.

The primary outcome is the rate of non union in each group at 3 months. Secondary outcomes are Constant-Murley and DASH scores at 3 months and 9 months following injury.

All of the analyses will use the intention-to-treat principle. The primary outcome will be compared between the conservative and operative groups using generalised linear regression models both unadjusted and adjusted for centre (the stratification factor), age, fracture classification and ASA grade. Estimates of the difference in proportions and odds ratio with associated 95% confidence intervals will be obtained.

The Constant-Murley and DASH outcomes will be compared between groups using analysis of covariance. This will allow for a baseline score as suggested through measurements recorded for the other normal limb. The assumptions of this approach will be checked using summary information and graphs. If assumptions are not met, appropriate data transform or non parametric methods will be considered. Multiple regression models will be used to investigate the intervention effect after adjustment for centre, age, fracture classification and ASA grade.

Where outcome data are missing, a check of the characteristics of those with and without the outcome of interest will be carried out to ensure that missing data have not biased a comparison based on complete data. All analyses will be carried out using Stata.

#### Trial timeline

Following a successful initial application for a British Society of Elbow and Shoulder Surgery primer grant, a period of 1 year for trial preparation, obtaining ethics approval, and Research and Development approval, a 6 month period establishing and piloting of all trial materials and processes was commenced at the Royal Free Hospital in December 2007, the official start date for general recruitment was July 2008. The award of a BUPA foundation grant allowed the appointment of a dedicated researcher in 2010 and the subsequently inclusion on National Institute of Health Research portfolio of clinical trials has allowed expansion of the trial sites. Currently there are 4 sites recruiting, and 4 further sites are planned, 2 of which have approval in place. The further planned recruitment period is 18 months, and completion of follow-up 9 months later. Study completion, which includes submission of the draft trial report to the funders for publication is scheduled for December 2012. The date of registration of the trial is 07/09/2006. The date the first patient was recruited is 18/12/2007.

#### Dissemination of trial findings

We shall disseminate our findings through relevant local, national and international conferences and peer-reviewed publications. Reflecting the collaborative basis of this research, all active contributors will be named and credited in the main report.

#### Trial management

The day to day management of the project is the responsibility of the Trial Management Group:

• Clinical co-ordination: Philip Michael Ahrens (Chief Investigator).

• Trial management: Emily Tims (Trial Co-ordinator, Royal Free Hospital), Suzanne Hodgson, Clinical Trial Co-ordinator (University College London), replaced by Temi Giwa (Univerity College London), Clinical Trial Co-ordinator, August 2010.

• Methodological support: Julie Barber (University College London).

The Trial Steering Committee is at the University College London Clinical Trials Unit. Specifically assigned to the Clavicle trial are a Statistician (Dr Julie Barber), a trial co-ordinator (Temi Giwa) and a Data Manager (Ms Kadija Rantell).

## Discussion

This article describes version 2.0 (01/09/10) of the protocol for a multicentre randomised controlled trial of conservative management versus ORIF for mid-shaft clavicle fractures in adults. Various adjustments, all approved by ethics, have been made to the original protocol approved by ethics. Many changes were clarifications in wording. A few others were in response to feedback, and to overcome practical barriers in trial recruitment. All changes, which have been fully documented, are likely to improve the prospects of the trial successfully meeting its aims.

## Competing interests

The authors declare that they have no competing interests.

## Authors' contributions

All of the authors contributed to the design and development of the trial protocol. UGL and PMA were responsible for writing this manuscript. All authors read and approved the final manuscript.
